# Low sclerostin levels after long-term remission of acromegaly

**DOI:** 10.1007/s12020-021-02850-7

**Published:** 2021-08-26

**Authors:** Kim M. J. A. Claessen, Iris C. M. Pelsma, Herman M. Kroon, Antoon H. van Lierop, Alberto M. Pereira, Nienke R. Biermasz, Natasha M. Appelman-Dijkstra

**Affiliations:** 1 Department of Medicine, Division of Endocrinology, and Center for Endocrine Tumors Leiden, Leiden, The Netherlands; 2 Department of Medicine, Division of Endocrinology, and Center for Bone Quality, Leiden, The Netherlands; 3 Department of Radiology, Leiden University Medical Center, Leiden, The Netherlands

**Keywords:** Sclerostin, Acromegaly, Insulin-like growth factor-1, Vertebral fractures, Osteoporosis, Bone microstructure

## Abstract

**Purpose:**

Bone health is compromised in acromegaly resulting in vertebral fractures (VFs), regardless of biochemical remission. Sclerostin is a negative inhibitor of bone formation and is associated with increased fracture risk in the general population. Therefore, we compared sclerostin concentrations between well-controlled acromegaly patients and healthy controls, and assessed its relationship with bone mineral density (BMD), and VFs in acromegaly.

**Methods:**

Seventy-nine patients (mean age 58.9 ± 11.4 years, 49% women) with controlled acromegaly, and 91 healthy controls (mean age 51.1 ± 16.9 years, 59% women) were included. Plasma sclerostin levels (pg/mL) in patients were measured with an ELISA assay, whereas in controls, serum levels were converted to plasma levels by multiplication with 3.6. In patients, VFs were radiographically assessed, and BMD was assessed using dual X-ray absorptiometry.

**Results:**

Median sclerostin concentration in controlled acromegaly patients was significantly lower than in healthy controls (104.5 pg/mL (range 45.7–234.7 pg/mL) *vs* 140.0 pg/mL (range 44.8–401.6 pg/mL), *p* < 0.001). Plasma sclerostin levels were not related to age, current growth hormone (GH) or insulin-like factor-1 (IGF-1) levels, gonadal state, treatment modality, remission duration, or BMD, VF presence, severity or progression.

**Conclusion:**

Patients with long-term controlled acromegaly have lower plasma sclerostin levels than healthy controls, as a reflection of decreased osteocyte activity. Further longitudinal studies are needed to establish the course of sclerostin during different phases of disease and its exact effects in acromegalic osteopathy.

## Introduction

Vertebral fractures (VFs) are a common complication of acromegaly, and contribute to the frequently observed thoracic kyphosis and high prevalence of back pain in these patients [1–3]. Prevalence of radiographic VFs is up to 40% during active disease, with even higher numbers after long-term follow-up in controlled patients (~60%) [4]. Several prospective studies revealed that VFs not only occur during the active phase of disease but additionally progress during prolonged follow-up in remission with male sex, hypogonadism and preexistent fractures as main risk factors for deterioration [2, 5, 6].

The pathogenesis of acromegalic osteopathy is multi-factorially determined. During active disease, supraphysiological growth hormone (GH) and insulin-like growth factor-1 (IGF-1) levels increase endocortical bone turnover in favor of bone formation, as reflected by biochemical bone markers and bone histomorphometry, leading to higher cortical BMD in the presence of stable trabecular bone mass [4, 7–11]. Several reports show that, despite a decrease in bone turnover, the GH-induced anabolic effects on bone mass sustain after prolonged remission in a large subset of patients [4, 12–14]. Nevertheless, the increased GH-induced bone remodeling is considered to be harmful because of persistent structural changes in trabecular bone with loss of trabecular connectivity.

However, recent studies reported that skeletal fragility in acromegaly cannot solely be explained by alterations in bone mass, but is mainly the consequence of GH/IGF-1-induced alternations in bone microstructure that impair bone quality [8, 11]. Both histomorphological and imaging studies have shown increased cortical thickness and porosity with increased trabecular widening, characteristics that persist after disease remission as indication of irreversible damage [15–18]. These findings were confirmed by reports of significantly lower dual energy X-ray (DXA)-derived trabecular bone scores in both active and controlled acromegaly patients, and are also underlined by the observed lower bone strength in controlled acromegaly, as measured by micro indentation [19–21]. Next to direct GH-/IGF1-induced effects, other factors such as vitamin D deficiency, hypogonadism and aggressive replacement of hypopituitarism, especially of glucocorticoids, contribute to the skeletal fragility of patients with acromegaly [1]. Until now, the exact underlying mechanisms for these long-lasting changes in bone quality remain unknown.

Sclerostin, a protein encoded by the SOST gene produced by osteocytes, is a negative inhibitor of bone by antagonizing wingless/integrated (Wnt) signaling preventing osteoblast differentiation [22, 23]. As bone turnover is affected in acromegaly, sclerostin could be an interesting player in the alteration of bone quality in acromegaly. During the last year, several research groups investigated sclerostin levels in relationship to disease activity and fractures in acromegaly, showing contradictory results [24–26]. Not only the level of sclerostin values differed significantly across studies when compared between patients in different acromegaly phases and healthy controls, also the direction of associations between sclerostin and GH/IGF-1 levels was highly variable. Until now, sclerostin levels could not be directly linked to VF presence [24–26]. Since prospective studies are lacking at this moment, the exact role of sclerostin in acromegalic bone disease remains unknown.

The main aim of the current study was to investigate plasma sclerostin levels in a unique cohort of long-term biochemically controlled acromegaly patients in comparison to healthy controls, and, within patients with acromegaly, to assess the potential relationship between sclerostin, BMD and VFs.

## Methods

### Study design

This cross-sectional study compared well-controlled acromegaly patients with healthy controls with an additional prospective part of the study describing a subset of acromegaly patients radiographically followed over 9 years (*see below*). In both patients and controls, plasma sclerostin levels were measured according to the same protocol. In patients, sclerostin levels were additionally studied in relationship to parameters reflecting GH/IGF-1 activity, bone turnover, BMD measurements and radiographic VFs. The study was approved by the Medical Ethics Committee of the Leiden University Medical Center (LUMC), and all included subjects gave written informed consent.

### Acromegaly patients

In 2007, all patients of the outpatient clinic of the LUMC with biochemically controlled acromegaly for ≥2 years were invited for a prospective study on musculoskeletal complications of acromegaly. On that occasion, 89 patients with controlled acromegaly were included for the baseline visit, as published previously [2, 27, 28]. Data of this baseline visit were used for the current study, resulting in the inclusion of 79 patients (89%) with available plasma sclerostin levels. In all patients, standardized questionnaire concerning demographic data and medical history were completed, postabsorptive blood samples were taken, and conventional spine radiographs were performed. In a subset of patients, we had available additional 9-year radiographic follow-up data on the VF course over time; these data have been published recently [27]. In another subset of patients, additional BMD measurements were available.

Details on the clinical follow-up and different treatment modalities of acromegaly in our center were previously described [2, 28]. A subset of patients was treated for osteoporosis at the discretion of the treating physician according the Dutch national guidelines [29]. At the time of the study visit, ten (13%) patients received bisphosphonates, and 20 (25%) and sixteen patients (20%) calcium and vitamin D supplementation, respectively (see Table 1).

**Table 1:** Clinical characteristics of controlled acromegaly patients and healthy controls

Clinical characteristics	Patients (*N* = 79)	Controls (*N* = 91)	*P* value
Age (years)	59.9 ± 11.4	51.1 ± 16.9	<0.001
Sex, female (*n*(%))	39 (49%)	54 (59%)	0.193
BMI (kg/m^2^)	28.3 ± 4.6	25.3 ± 4.3	<0.001
Treatment (*n*(%))^a^			
Surgery only	45 (57%)	NA	
RT only	1 (1%)		
Surgery + RT	10 (13%)		
SMS analogs			
Primary	5 (6%)		
Following surgery	15 (19%)		
Following RT	1 (1%)		
Following surgery + RT	2 (3%)		
Disease duration (years)	9.0 ± 7.4	NA	
Duration of remission (years)	14.6 ± 5.9	NA	
Pre-treatment GH (µg/L)	35.7 ± 46.4	NA	
IGF-1 SD scores			
Pre-treatment	7.5 ± 4.8	NA	
Actual	0.45 ± 1.53		
Hypopituitarism (n(%))			
Corticotrope failure	19 (24%)	NA	
Thyreotrope failure	16 (20%)		
Gonadotrope failure^b^	45 (56%)		
GHD	11 (14%)		
Vitamin D25(OH) (nmol/l)	70.2 ± 23.9	NA	
PTH (pmol/l)	6.1 ± 2.8	NA	
Bone markers			
P1NP (ng/mL)	37.9 ± 22.2	NA	
β-crosslaps (ng/mL)	0.34 ± 0.21	NA	

Values are depicted as mean (SD) unless stated otherwise.

*GH* growth hormone, *IGF-1* insulin-like growth factor-1, *BMI* body mass index, *RT* radiotherapy, *SMS* somatostatin analogs, *GHD* growth hormone deficiency, *BMD* bone mineral density, *PTH* parathyroid hormone, *P1NP* procollagen type 1 amino-terminal propeptide, *NA* not applicable.

^a^Two patients (2.5%) were co-treated with Pegvisomant.

^b^Including natural menopause (*N* = 36 (46%)) and hypogonadotropic hypogonadism (*N* = 9 (11%)).

### Healthy controls

We included an existing cohort of 91 healthy controls with available sclerostin measurements, derived from the same geographic area as the acromegaly patients. All controls had a normal gonadal status, normal serum calcium concentration, bone turnover and renal function. None of the control subjects were treated with bisphosphonates or glucocorticoids. Part of these control data has been published previously [30].

### Acromegaly disease parameters

Disease activity was assessed at least annually, by assessment of fasting serum GH and IGF-1 levels, and additional periodical oral glucose tolerance tests in non-medically treated patients. For all treatment modalities, patients were considered to be in remission when IGF-1 levels (based on SD scores (SDS)) and glucose-suppressed GH levels were normal. Active disease duration was calculated using the estimated date of disease onset and the date of IGF-1 normalization following treatment, whereas remission duration was based on the date of biochemical remission to the date of the study visit [2, 28].

### Assessment of endocrine function

Hypopituitarism was defined as a clinically significant hormone deficiency in ≥1 pituitary axis, which was adequately treated with hormonal supplementation according to previously described definitions [31, 32]. GH deficiency (GHD) was only assessed on clinical or biochemical suspicion.

### Biochemical assays

Different assays for GH and IGF-1 were used over time during follow-up of the patients. For GH, a radioimmunoassay (RIA) was used before 1992 (Biolab, Serona, Coissins, Switzerland), followed by a 22-kDA GH protein immunofluorometric assay (Wallac, Turku, Finland) from 1992 to 2005. From 2005 to 2017, GH was measured using a nationally harmonized GH assay on the Immulite 2500/2000XPi immunoanalyser (harmonization factor: 1.02) [33] and from 2017 onwards, the IDS-iSYS analyzer is used. For the conversion of µg/L to mU/L, values were multiplied by 2.6.

Serum IGF-1 concentrations were measured by a RIA until 2005 (Incstar, Stillwater, MN, USA), followed by an immunometric technique using an Immulite 2500 system (Diagnostic Products Corporation, Los Angeles, CA, USA) from 2005-2017. Since 2017, IGF-1 is measured on IDS-iSYS immunoanalyser. More details of these assays were described before [2]. IGF-1 levels are expressed as SDS, using age- and sex-dependent lambda-mu-sigma smoothed reference curves [34, 35].

Markers of bone turnover, being aminoterminal propeptide of type I procollagen (P1NP, bone formation) and β-crosslaps (bone resorption), were measured by the E-170 system (Roche BV, Woerden, The Netherlands). Reference ranges were ULN < 59 ng/mL for pre-menopausal women and men, and <76 ng/mL for postmenopausal women for P1NP, and ULN < 0.573 ng/mL for premenopausal and <1.008 ng/mL for postmenopausal women, <0.704 ng/mL for men until 70 years and <0.854 ng/mL for men >70 years old for β-crosslaps. All analyses were performed according to the manufacturers’ protocol. Serum levels of parathyroid hormone (PTH) were measured using Immulite 2500 (Siemens Diagnostics, Breda, The Netherlands).

In patients, only plasma EDTA samples were available for sclerostin measurement, retrieved from archived material that had been stored at −80 °C, whereas in controls, sclerostin was measured in serum. Sclerostin was measured in pg/mL by an electrochemiluminescence assay (MSD 96-well MULTI-ARRAY Human Sclerostin Assay; Meso Scale Discovery, Gaithersburg, MD, USA; detection limit of ±1 pg/mL), as previously described [36]. For the conversion to plasma levels, serum sclerostin values were multiplied by 3.6, based on previous literature showing that sclerostin levels in plasma are 3.6 ± 1.0 times higher than serum levels with a high correlation between serum and plasma levels (*r* = 0.91, *p* = 0.001) [37]. Using this assay, sclerostin was previously shown to be undetectable in the serum of all 19 patients with sclerosteosis tested [36]. In every run, a control sample from a patient with sclerosteosis and 3 control samples with different values were included. Results of these measurements were highly reproducible (mean values in pg/mL [CV] in 18 runs with different batches were as follows: 20.9 [7.4%], 37.7 [6.2%], and 106.3 [7.4%]).

### Radiographic VF assessment and evaluation of non-vertebral fractures

In all patients, conventional lateral thoracic and lumbar spine radiographs were performed according to a standardized protocol, with the film centralized on Th7 and L3, respectively. All radiographs were assessed for VF presence in vertebrae Th4 to L4 according to the validated Genant’s scoring method [38]. Grade 1 (mild fracture) was defined as 20–25% reduction in anterior, middle and/or posterior height; grade 2 (moderate fracture) as 25–40% reduction in anterior, middle and/or posterior height; grade 3 (severe fracture) >40% reduction in anterior, middle and/or posterior height. Wedge, biconcave and concave fractures were distinguished. Radiographs were scored blinded for any patient characteristics by two scorers in consensus (K.C. and H.K.), of whom the latter is a very experienced musculoskeletal radiologist (H.K.). Six individual vertebrae were excluded because of pre-existing pathology. The intra-observer variability was good with an intra-correlation coefficient (ICC) of 0.883.

In the subset of patients with available 9-year follow-up radiographs, spine radiographs of both study visits were assessed simultaneously in chronological order by the same scoring team (H.K. and K.C.) [39]. VF progression was defined as a new VF or a ≥1-point increase in Genant score of a preexisting fracture. Part of these VF data have been published previously [27, 28]. Further details on radiographic assessment and scoring are reported elsewhere [2, 28].

In addition, as a measure of VF severity, a spinal deformity index (SDI) was calculated as the sum of the VF grades according to the Genant scoring method of all 13 scored vertebrae (Th4 to L4) within a patient, with SDI ranging from 0 to 39 [40].

Using a structured self-reported questionnaire, prevalence of non-vertebral fractures after inappropriate trauma was assessed, without inclusion of radiographic fracture data.

### BMD measurements

BMD values were only available in a subset of patients and have been published for the original cohort before [28]. BMD measurements were not available in controls. BMD was measured at the lumbar spine (L1-L4) and total hip using DXA (Hologic QDR 4500, Hologic Inc., Waltham, MA, USA), equipped with reference values based on the National Health and Nutrition Examination Survey (NHANES III). Osteopenia and osteoporosis were defined using World Health Organization (WHO) criteria (T-scores between −1.0 and −2.5, and ≤−2.5, respectively) [41].

### Statistical analysis

Data were analyzed using SPSS version 26.0 (SPSS Inc., Chicago, IL). Values are reported as mean ± SD, unless otherwise stated. Median sclerostin levels were compared between patients and controls using a linear regression model, reporting a β ± standard error (SE), adjusting for age and BMI, since these factors were previously published to correlate positively with serum sclerostin levels [42–44], and, moreover, these parameters significantly differed between patient and controls (Table 1). Correlations between sclerostin levels and acromegaly-specific disease markers were assessed by Pearson’s correlation. Median sclerostin concentrations were compared between patients with and without VFs using a non-parametric test. A logistic regression model was used for evaluation of the relationship between sclerostin and the presence and progression of VFs with corrections for age, sex, BMI and current IGF-1 levels. A *p* value < 0.05 was considered significant.

## Results

### Clinical characteristics

Clinical characteristics of the 79 included controlled acromegaly patients (mean age 58.9 ± 11.4 years, 49% female) and 91 healthy controls (mean age 51.1 ± 16.9 years, 59% female) are shown in Table 1. Mean remission duration was 14.6 ± 5.9 years, with solely two patients having a remission duration of <5 years. Forty-five patients were cured by surgery alone (57%), ten patients by combined surgery and postoperative radiotherapy (13%), and 23 patients (29%) were pharmacologically controlled by long-acting SMS analogs, either as primary or secondary treatment. Two patients (3%) received Pegvisomant co-treatment. During follow-up, two patients showed biochemical evidence of disease recurrence, as reflected by IGF1 SDS > 2.5 accompanied by abnormal glucose-suppressed GH values, resulting in initiation of adjuvant SMS analog treatment in both patients. Mean current IGF-1 SDS was 0.45 ± 1.53.

P1NP and β-crosslaps were within the reference range for all patients, overall being relatively low, except for slightly elevated P1NP levels in four postmenopausal women. Sixteen patients (20%) received calcium supplementation, twelve patients (15%) received vitamin D supplementation and ten patients (13%) were treated with bisphosphonates.

### Comparison of sclerostin concentrations with healthy controls

**Fig. 1:**
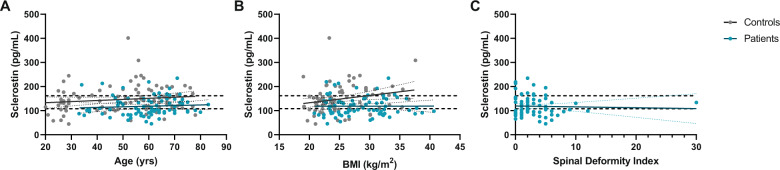
Individual plasma sclerostin levels *versus* age, BMI and spinal deformity index for biochemically controlled acromegaly patients and healthy controls. Individual plasma sclerostin levels versus age (**A**), BMI (**B**) and spinal deformity index (**C**) are depicted for controlled acromegaly patients (blue dots) and controls (gray dots). The spinal deformity index was only calculated for patients. The upper and lower dashed lines represent, respectively, the upper limit of plasma sclerostin levels of 162 pg/mL and the lower limit of 108 pg/mL. Within patients, there were no clear correlations between plasma sclerostin levels and age, BMI or SDI, respectively (*i.e*. *r* = 0.074, *p* = 0.515 for age, *r* = 0.03, *p* = 0.979 for BMI and *r* = −0.036, *p* = 0.751 for SDI), whereas in controls, plasma sclerostin levels correlated positively with BMI (*r* = 0.409, *p* < 0.001), but not with age (*r* = 0.140, *p* = 0.186). BMI body mass index, SDI spinal deformity index

In controls, median plasma sclerostin concentration was 140.0 pg/ml (range 44.8–401.6 pg/ml), with individual levels plotted in Fig. 1. Within controls, sclerostin was positively associated with BMI (*r* = 0.409, *p* < 0.001), but not with age (*r* = 0.140, *p* = 0.186). Median sclerostin concentrations were comparable between female and male controls (131.3 pg/ml (range 44.8–309.3 pg/ml) *vs* 154.6 pg/ml (range 64.2–401.6pg/ml), *p* = 0.114).

**Fig. 2:**
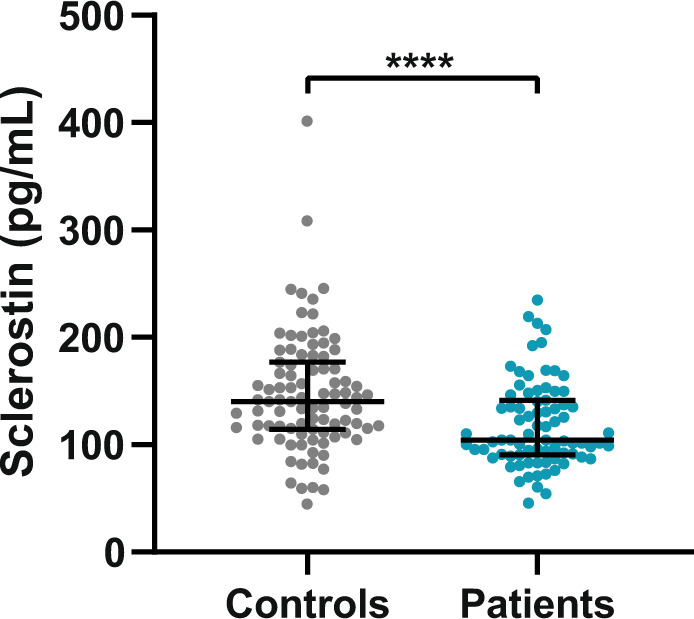
Median sclerostin levels of controlled acromegaly patients *vs* healthy controls. The bars represent median plasma sclerostin concentrations (pg/mL), with 95% confidence intervals. It should be noted that in patients plasma levels were measured directly, whereas in controls serum levels were measured, which were in turn converted to plasma levels by a multiplication factor for 3.6, as described in the methods. Plasma sclerostin levels were significantly higher in healthy controls than in long-term controlled acromegaly patients, after adjustments for age, sex and BMI (*p* < 0.001)

When compared to controls, acromegaly patients had significantly lower sclerostin levels, after adjustments for age and BMI (adjusted β = −36.0 ± 8.1, *p* < 0.001), with comparable results for both sexes (Fig. 2). Results did not change upon exclusion of the ten patients treated with bisphosphonates (adjusted β = −36.2 ± 8.6, *p* < 0.001), with comparable results between males and females.

### Sclerostin concentrations in patients with controlled acromegaly

Median sclerostin concentration in controlled acromegaly patients was 104.5 pg/mL (range 45.7–234.7 pg/mL), showing comparable levels between men and women (110.0 pg/mL (range 70.0–219.2 pg/mL) in men *vs* 101.6 pg/ml (range 45.7–234.7 pg/mL) (*p* = 0.430) in women). Plasma sclerostin concentrations for individual patients in relationship to age and BMI were plotted in Fig. 1A, B, showing no significant correlation between sclerostin and either age or BMI (*r* = 0.074, *p* = 0.515 for age, and *r* = 0.03, *p* = 0.979 for BMI).

We did not find any significant correlations between sclerostin levels and, respectively, pre-treatment GH or IGF-1 levels, current IGF-1 levels, treatment modality, use of bisphosphonates, hypogonadism or duration of remission, although for the latter, it should be noted that only two patients had a remission duration <5 years (*data not shown*). Moreover sclerostin levels were not significantly correlated to P1NP or β-crosslaps levels, independently of the use of bisphosphonates (*data not shown*).

#### Sclerostin concentrations in relation to vertebral and non-vertebral fractures

In this long-term controlled acromegaly cohort, 61 patients (77%) showed ≥1 radiographic VF (mean VF number per patient: 2.2 (range 1–9 fractures)), of whom 17 patients (21.5%) had ≥1 grade 2 or 3 fracture. Almost all fractures were of the anterior wedge type, showing a bimodal fracture pattern with peaks at the levels Th7 to Th9, and Th11 to L1. BMD values were comparable between patients with and without VFs: mean BMD lumbar spine 0.99 ± 0.17 g/cm^2^ in patients with VFs *vs* 1.02 ± 0.15 g/cm^2^ in patients without VFs (*p* = 0.655) and mean BMD total hip 0.94 ± 0.17 g/cm^2^ in patients with VFs *vs* 0.92 ± 0.10 g/cm^2^ in patients without VFs (*p* = 0.709). Patients with and without VFs were comparable with respect to hypogonadism prevalence. In the subset of 25 patients with available 9-year follow-up radiographs, VF progression was observed in 10 patients (40%).

Median sclerostin concentrations were comparable between patients with and without VFs (104.5 pg/ml (range 45.7–234.7 pg/ml) *vs* 96.2 pg/ml (range 65.6–219.2 pg/ml), *p* = 0.070), without a change upon analyzing patients with grade 2 or 3 fractures only (*data not shown*). Also after adjustments for age, sex, BMI and current IGF-1 levels, plasma sclerostin levels were not related to either the VF presence, VF number or its progression over time (*data not shown*). In addition, plasma sclerostin levels were not correlated to the SDI (*r* = −0.036, *p* = 0.751; Fig. 1C).

A subset of patients experienced non-vertebral fractures after inappropriate trauma, based on self-reported questionnaires, including 2 hip fractures, 5 lower limb fractures, and 13 other fractures on different localizations. Sclerostin levels were comparable between patients with and without a history of non-vertebral fractures (*p* = 0.359).

#### Sclerostin concentrations in relation to BMD

In the subset of 49 patients (62%) with available BMD values, mean BMD at the lumbar spine was 1.00 ± 0.16 g/cm^2^ and mean ***T***-score was −0.64 ± 1.44. Mean BMD at the total hip was 0.94 ± 0.16 g/cm^2^ and mean ***T***-score was −0.84 ± 2.21. According to the WHO criteria, resp. 15 and 6 patients had osteopenia and osteoporosis at the lumbar spine, and resp. 12 and 10 patients had osteopenia and osteoporosis at the total hip. Sclerostin concentrations were not correlated to BMD values, adjusted for age, sex and BMI, or presence of osteoporosis (*data not shown*).

## Discussion

This study demonstrated significantly lower plasma sclerostin levels in patients with long-term well-controlled acromegaly compared to healthy controls. BMI correlated to sclerostin levels in controls, but not in acromegalic patients, and there was no significant correlation with age. Within controlled acromegaly patients, we could not detect a significant relationship between sclerostin levels and parameters reflecting GH/IGF-1 activity. In addition, sclerostin levels were not significantly associated with bone turnover, BMD, VF presence or 9-year VF progression.

The protein sclerostin is produced mainly by osteocytes, and antagonizes the Wnt signaling pathway in osteoblast lineage cells, thereby negatively regulating bone formation [22, 23]. The role of sclerostin in bone homeostasis has been intensively discussed during the last decade, based on insights gained from studies in the very rare monogenic disorders sclerosteosis and van Buchem disease, in which patients have a congenital sclerostin deficiency due to mutations affecting the SOST gene. In the general population, most studies show an association between increased serum sclerostin levels and declining BMD values [45]. Artificial lowering of sclerostin levels in postmenopausal women with osteoporosis results in a reduction of bone resorption and increase in bone formation, eventually increasing BMD and thereby reducing fracture risk [46, 47].

Recent research has shown that in acromegaly especially the (partially irreversible) altered bone microstructure is responsible for the high fracture rate, rather than changes in bone quantity [8, 11]. Exact underlying mechanisms, however, have not been fully elucidated. Since sclerostin and bone fragility are linked in the general population, sclerostin became a potential target for therapy, also in acromegaly.

Two recently published cross-sectional studies investigated sclerostin levels in controlled acromegaly before [25, 26], although with significantly smaller patient numbers (18 and 12 controlled patients, respectively), both reporting comparable serum sclerostin levels between patients and healthy controls [25, 26]. In this respect, several important differences between our study and the two other reports in controlled patients have to be mentioned, probably (partially) explaining the inconsistency of results. In our opinion, the most important difference between the studies is the duration of biochemical disease remission. In our study, all patients were in remission of acromegaly for a mean of 14.6 ± 5.9 years, reflecting longstanding disease remission, whereas in the study of Silva et al. mean remission duration was only 3 years (range 1–14 years), and Uygur et al. only reported the proportion of patients in remission, but no remission duration. Since osteocyte apoptosis is elevated in bone with high rates of modeling or remodeling [48], which is the case in active acromegaly, osteocyte apoptosis is upregulated during active disease. This first phase of osteopathy is followed by a chronic phase that starts after achievement of biochemical remission. However, the exact timing when this chronic remission phase is achieved is unclear, but it takes some time to achieve a new hormonal balance (*see below*). Also the duration of active disease might be of influence on this timing.

Available literature on sclerostin levels during active acromegaly also reports contradictory data, showing inconsistent associations between sclerostin and GH/IGF-1 levels, ranging from positive to negative correlations [24–26]. However, the studied cohorts were very diverse (Table 2), especially with respect to age, VF prevalence and menopausal state, making it hard to draw firm conclusions. Since there is evidence for both younger age and estrogen to be associated with lower sclerostin levels, these parameters are likely to play a role in the inconsistency of results [42, 49].

**Table 2:** Overview of current literature on sclerostin levels in acromegaly

Author	Journal (yr)	Study design	Acromegaly patients	Controls	Sclerostin assay	Sclerostin levels	Sclerostin correlations	BMD	VFs
Pekkolay et al.	J Clin Endocrinol Metab (2020)	Case-control	Active *N* = 30 47 yr, 53%F	Healthy controls *N* = 30 45 yr, 43%F Matched for age/sex/BMI	ELISA Serum (ng/mL)	29.95 ng/mL patients *vs* 22.44 ng/mL controls (*p* < 0.05)	Positive correlation with GH&IGF1 No correlation with hypogonadism	NA	NA
Silva et al.	J Clin Endocrinol Metab (2021)	Case-control	Active *N* = 12/remission *N* = 18 40 yr, 100%F (premenopausal) Remission duration 3 yr (1 = 14 yr) No hypopituitarism	Healthy controls *N* = 53 40 yr, 100%F (premenopausal) Matched for age/sex/BMI	ELISA Serum (pg/mL)	17.2 pg/mL active *vs* 28.4 pg/mL controls (*p* = 0.041) 17.2 pg/mL active *vs* 30.9 pg/mL remission (*p* = 0.021) 30.9 pg/mL remission *vs* 28.4 pg/mL controls (NS)	Positive correlation with active disease duration Negative correlation with IGF-1&osteocalcin	NA	VF prevalence 30%^a^ 29.5 pg/mL VF *vs* 22.1 pg/mL no VF (NS)
Uygur et al.	Endocrine (2021)	Cross-sectional	Active *N* = 54/remission *N* = 16 46 yr, 49%F	Healthy controls *N* = 70 46 yr, 44%F Not matched	ELISA Serum (ng/mL)	10.4 ng/mL active *vs* 11.1 ng/mL remission (NS) 10.5 ng/mL all patients *vs* 11.3 ng/mL controls (NS)	No correlation with age/GH/IGF-1	NA	VF prevalence 73%^a^ 11.5 ng/mL VF *vs* 8.8 ng/mL no VF (NS)
Claessen et al.		Cross-sectional with prospective part	Remission *N* = 79 59 yr, 49%F Remission duration 14.6 ± 5.9 yr	Healthy controls *N* = 91 51 yr, 59%F Not matched	ELISA Plasma (pg/mL)	104.5 pg/mL remission *vs* 140.0 pg/mL controls (*p* < 0.001)	No correlation with age/GH/IGF1& hypogonadism	No correlation with BMD	VF prevalence 77%^a^ 104.5 pg/mL VF *vs* 96.2 pg/mL (*p* = 0.07) No association with VF progression or SDI

*Yr* year, *N* number of patients, *F* female, *GH* growth hormone, *IGF-1* insulin-like growth factor-1, *BMD* bone mineral density, *VFs* vertebral fractures, *NA* not assessed, *SDI* spinal deformity index

^a^VF grading according to Genant scoring method

With respect to the association between sclerostin and fractures, we did not detect a clear association with VF presence, neither with VF progression in the prospective part of our study. These results correspond with the findings of two recent reports, in which sclerostin levels could also not be (directly) linked to VFs [25, 26] (Table 2), likely because of the multifactorial nature of fracture risk in acromegaly. Getting all available data together, we may conclude that in the heterogenous cohorts of acromegaly patients in which sclerostin has been studied to date, sclerostin values are highly variable with unknown clinical significance of this variability. However, we assume that acromegaly activity and duration of biochemical remission are important determinants which altogether with other factors, including age, menopausal state, and hypopituitarism may explain the observed differences in sclerostin levels between the studies.

**Fig. 3:**
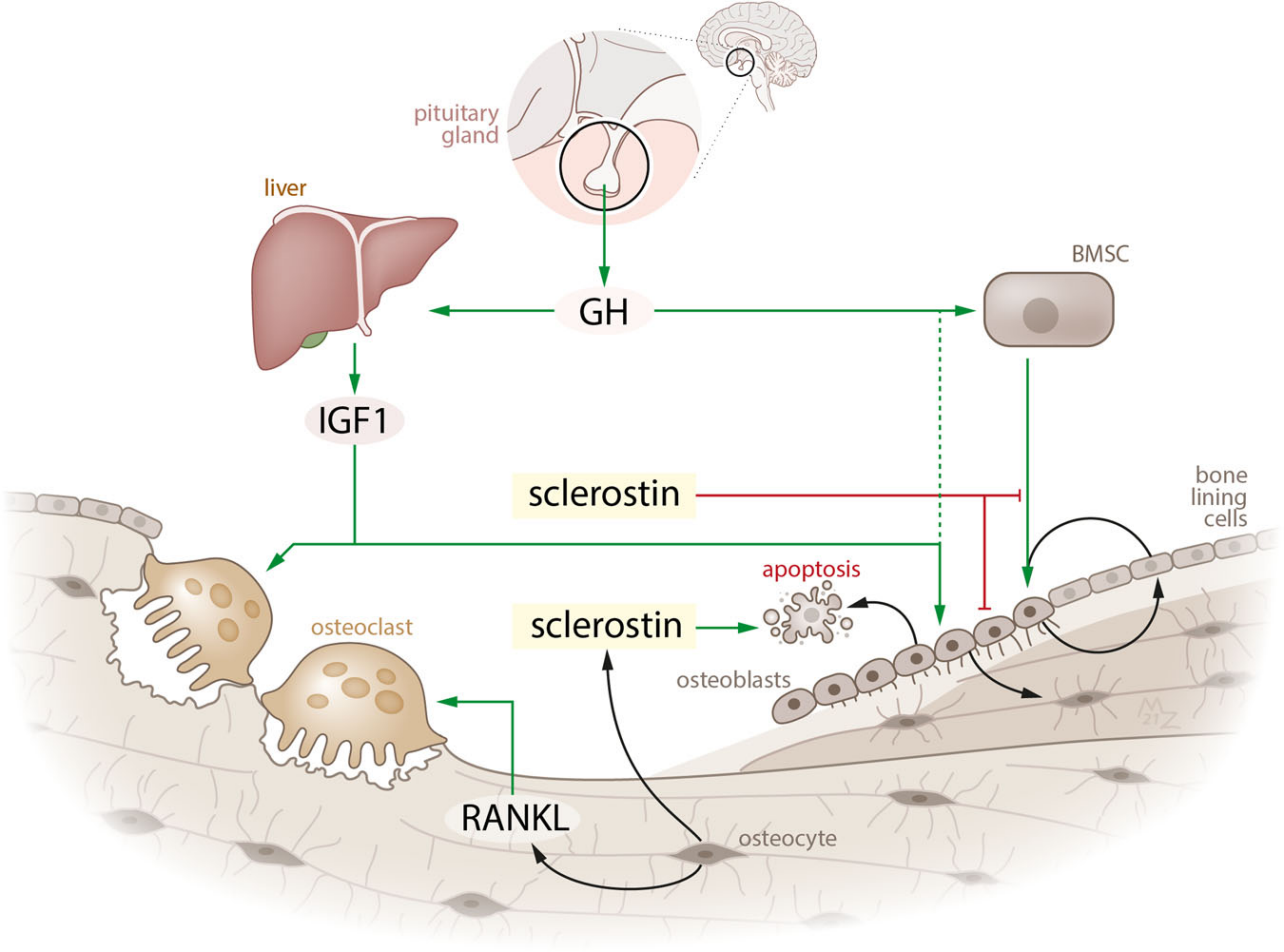
Hypothetical model of the role of sclerostin in the pathophysiology of skeletal fragility in patients with acromegaly. This figure shows the different actions of GH and IGF-1 on bone in patients with acromegaly (during active disease), and the hypothetical effects/interactions of sclerostin in acromegalic bone disease. During active disease, supraphysiological GH and IGF-1 levels increase endocortical turnover with increasing bone remodeling sites, and we hypothesize that overall sclerostin production is thereby increased as reflection of increased osteocyte activity. In turn, these higher sclerostin levels inhibit bone formation and increase its resorption. After achievement of GH/IGF-1 control, bone turnover normalizes/decreases again, thereby decreasing sclerostin production. This may have positive anabolic effects on bone, probably as a compensatory mechanism to increase bone turnover. Green arrow, stimulating effect; Red arrow, inhibitive effect. GH, growth hormone; IGF-1, insulin-like growth factor-1, RANKL, receptor activator of nuclear factor kappa-Β ligand

Therefore, based on current evidence, we hypothesize that three different phases of osteopathy can be identified, and that sclerostin levels, although there might be large interindividual variability, are likely to differ between these phases. First, during active acromegaly, when patients are exposed to supraphysiological GH and IGF-1 levels, there is increased endocortical turnover in the presence of an increased number of bone remodeling sites, but stable trabecular bone mass [7, 11]. Since bone formation and resorption are coupled, the increased bone turnover may result in overall increased sclerostin production by osteocytes [15], which may, in turn, lead to a decrease in bone formation and increased bone resorption. This first phase in which patients have highest fracture risk, is thereby likely to be characterized by high sclerostin levels, which corresponds to the results of Pekkolay and colleagues [24]. In this respect, GH and IGF-1 may have additional direct (stimulatory) effects on sclerostin production, and in the end osteocyte apoptosis, although this has not been studied before. In the second phase of osteopathy, ‘the early remission phase’ shortly after IGF-1 normalization, bone turnover decreases, as reflected by low bone turnover markers but also histomorphologically, showing significantly lower osteocyte and osteoblast numbers with decreased activity compared to active disease [15], but no steady state has been achieved. Finally, in the ‘chronic remission’ phase that starts after several years of biochemical remission, patients have reached a new hormonal steady state, which is likely to be accompanied by low sclerostin levels, as a reflection of decreased osteocyte activity. A hypothetical model of the place of sclerostin in acromegalic bone disease is shown in Fig. 3.

Another point to mention is that there is evidence for an irreversibly altered bone microstructure in acromegaly with a persistent very high VF prevalence despite remission, even with progression after longstanding biochemical control [15–18, 27, 28]. Therefore, the skeletal status in acromegaly differs from that in other endocrine diseases, such as primary hyperparathyroidism and Cushing’s disease, in which adequate control of the transient hormonal excess leads to (complete) bone recovery, accompanied by normalization of sclerostin levels [30, 50, 51].

Our study has several strengths and limitations. Clear strengths are the well-documented long-term follow-up of the patients, inclusion of a control group, and protocolled radiographic VF assessment by a team of experienced scorers. The main limitation of the study is the relatively small number of included acromegaly patients, probably resulting in a lack of power to detect a significant association between sclerostin levels and VFs. A second potential limitation is the difference in sclerostin measurement between patients and controls, since in patients we measured sclerostin in plasma samples, whereas in controls, sclerostin was measured in serum with subsequent conversion to plasma values in order to compare both groups. Although plasma and serum sclerostin levels are highly correlated, the conversion in controls could introduce small variations. Third, we included patients with bisphosphonates in our analyses. The effects of bisphosphonates on serum sclerostin levels are uncertain where some report an increase of serum sclerostin levels [52], whereas Anastasilakis et al. report a decrease of serum sclerostin levels after treatment initiation [53]. Finally, there is evidence that osteocytes, being sensitive to microdamage and shear stress due to tissue deformation [54], are able to express signaling molecules that regulate osteoblasts, osteoclasts, and probably also lining cells. The suppressive effect of sclerostin expression is thereby a strong indicator of whether an osteon is actively making bone at that time. In the light of this knowledge, serum sclerostin levels need to be interpreted with caution, since it is unknown whether these levels truly reflect sclerostin production and, moreover, whether they represent sclerostin concentrations at tissue level.

Future studies should include larger samples of patients, representing both genders and different types of treatment, and ideally with different phases of the disease, which are likely to have their own specific effects on bone architecture and micro-environment, as described above. Since within Europe, is has become possible to align policies in rare diseases in the European Reference Networks (ERN), we would like to call for international prospective studies addressing and integrating these questions in acromegaly-related osteopathy as international collaborative efforts enable access to larger populations, which is imperative for rare diseases like acromegaly. Furthermore, the optimal treatment strategy of the high fracture risk in acromegaly is unknown. Although data on the efficacy and safety of bone-modifying drugs in acromegaly are unavailable, patients with a low BMD and progressive VFs are likely to benefit from anti-resorptive drugs. This might be beneficial, with largest impact in the acute phase/during active disease when bone turnover is high, whereas in the remission state, drugs that potentially improve bone quality and osteocyte function might be the preferred option.

In conclusion, we demonstrated that plasma sclerostin levels were lower in patients with long-term controlled acromegaly compared to healthy controls, but could not directly link sclerostin levels to bone turnover, BMD or VFs. Since studies on sclerostin values in acromegaly show highly variable results, sclerostin is not likely to be the most important factor in acromegalic osteopathy, but could be a reflection of osteocyte activity. Further longitudinal studies are needed to establish the course of sclerostin levels during the different phases of acromegaly to elucidate its exact effects acromegalic bone disease.

## Data Availability

Available upon request.
